# Foul wind, spirits and witchcraft: illness conceptions and health-seeking behaviour for malaria in the Gambia

**DOI:** 10.1186/s12936-015-0687-2

**Published:** 2015-04-24

**Authors:** Sarah O’Neill, Charlotte Gryseels, Susan Dierickx, Julia Mwesigwa, Joseph Okebe, Umberto d’Alessandro, Koen Peeters Grietens

**Affiliations:** Department of Public Health, Institute of Tropical Medicine, Antwerp, Belgium; Medical Research Council, Fajara, The Gambia; London School of Tropical Medicine and Hygiene, London, UK; Faculty of Medical sciences, University of Antwerp, Antwerp, Belgium; School of International Health Development, Nagasaki University, Nagasaki, Japan; Partners for Applied Social Sciences (PASS) International, Tessenderlo, Belgium; School of Anthropology, University of Oxford, Oxford, UK

**Keywords:** Malaria, Symptom interpretation, Health-seeking behaviour, Treatment seeking behaviour, Elimination, Disease categories, Folk illnesses

## Abstract

**Background:**

As the disease burden in the Gambia has reduced considerably over the last decade, heterogeneity in malaria transmission has become more marked, with infected but asymptomatic individuals maintaining the reservoir. The identification, timely diagnosis and treatment of malaria-infected individuals are crucial to further reduce or eliminate the human parasite reservoir. This ethnographic study focused on the relationship between local beliefs of the cause of malaria and treatment itineraries of suspected cases.

**Methods:**

An ethnographic qualitative study was conducted in twelve rural communities in the Upper River Region and the Central River Region in the Gambia. The data collection methods included in-depth interviews, participant observation, informal conversations, and focus group discussions.

**Results:**

While at first glance, the majority of people seek biomedical treatment for ‘malaria’, there are several constraints to seeking treatment at health centres. Certain folk illnesses, such as *Jontinooje* and *Kajeje*, translated and interpreted as ‘malaria’ by healthcare professionals, are often not considered to be malaria by local populations but rather as self-limiting febrile illnesses – consequently not leading to seeking care in the biomedical sector. Furthermore, respondents reported delaying treatment at a health centre while seeking financial resources, and consequently relying on herbal treatments. In addition, when malaria cases present symptoms, such as convulsions, hallucinations and/or loss of consciousness, the illness is often interpreted as having a supernatural aetiology, leading to diagnosis and treatment by traditional healers.

**Conclusion:**

Although malaria diagnostics and treatment-seeking in the biomedical sector has been reported to be relatively high in the Gambia compared to other sub-Saharan African countries, local symptom interpretation and illness conceptions can delay or stop people from seeking timely biomedical treatment, which may contribute to maintaining a parasite reservoir of undiagnosed and untreated malaria patients.

## Background

Although malaria remains one of the world’s greatest killers of children [1], some malaria-endemic countries, including those in sub-Saharan Africa, have achieved remarkable success in reducing the disease burden [2-6]. In 2013, World Health Organization (WHO) projections showed that if the annual rate of decrease over the last 12 years was maintained, malaria mortality rates were expected to decrease by 52% in all ages and by 60% in children under the age of five by 2015 [7]. In the Gambia, the percentage of malaria-positive slides among patients attending health facilities has declined substantially over the last ten years [8], suggesting that malaria elimination may be possible, at least in some regions [9]. Nevertheless, malaria transmission has become more heterogeneous [10] with hotspots, i.e., locations with infected but asymptomatic individuals, able to maintain transmission.

Since the 1990s, WHO has recognized that, to maximize the impact of malaria control interventions, it is important to understand human behaviour within its sociocultural, economic, environmental, and political context [11-13]. Studies of treatment-seeking-behaviour and provider choice in malaria-endemic countries have identified key structural factors influencing interventions’ effectiveness, such as: availability and accessibility (distance to health centres, cost of transport), affordability (cost of treatment), resource-seeking and coping strategies (vulnerability) [14,15]. Additional factors that can be decisive for patients’ therapeutic itineraries are those linked to perceptions of disease and to ‘community understandings’ of malaria which often include biomedical elements but may diverge considerably from the biomedical model [14,16]. Clinical malaria symptoms are not always recognized or not necessarily associated with malaria, as is the case for convulsions, for example [15,17-22]. Certain clinical symptoms can be attributed to supernatural forces that necessitate the consultation of a traditional healer [14,16,19,20,23]. Even when people do know and understand the biomedical origin of malaria, it is still common to attribute the cause of the illness to supernatural forces, i.e.; in case of ambiguity during diagnosis, complications during the illness progression, and relapses. Treatment itineraries, therefore, frequently consist of a combination of biomedical and ‘traditional’ medicines [14-16,24].

Despite the good availability of diagnosis and treatment for malaria in the Gambia, it is still unclear why some individuals opt not to go for biomedical treatment but instead rely on home care or traditional healing. This ethnographic study focused on local beliefs of the cause of malaria and on treatment itineraries of suspected malaria cases, both uncomplicated and severe, in order to gain a better understanding of malaria patients’ health itineraries.

## Methods

### Study site and population

Ethnographic fieldwork was carried out between July and December 2013, and August and September 2014 in Fuladu East district (Upper River Region) in the villages of Ceesaykunda, Kolikunda, Kulluh Kouleh, Tinkinjo, Touba Tafsir, Keneba, Niji, Sare Gella, Tenkoly, and Sare Sillere and in Fuladu West district (Central River Region) in the villages of Boiram and Njoben. The study area is rural and inhabitants mostly live off subsistence farming (predominantly millet, maize, groundnut, and rice) and cattle herding as well as remittance from family members who regularly migrate to urban areas for trade or from relatives living abroad. Most inhabitants (90%) are Muslims, with 9% Christians and the rest practicing other religions. The main ethnicities represented in the study villages are Fula, Mandinka, Jahanka, Soninke (also locally referred to as Serahule), and Wolof.

Malaria transmission is moderate and highly seasonal, starting during the short rainy season (June-September), peaking from November until December/January. Malaria prevalence is heterogeneous, with the eastern part of the country having the highest prevalence [25]. In addition, according to the National Malaria Sentinel Surveillance Report [26], the prevalence of malaria is higher in children five to 14 years old (21.6%) than in children under five (7.8%), indicating a shift in vulnerability to older age groups.

### Research strategy

The data collection consisted of ethnographic qualitative methods:

**Participant observation** incorporates participating in everyday activities in the community setting, observing events in their usual context and carrying out informal conversations and interviews with a maximum variety of research subjects [27,28]. This method provides a more contextualized understanding of research questions when trying to grasp complex situations, such as health-seeking behaviour and how local people’s conceptions differ from public health theory. For this research, participant observation included sitting outdoors with research participants in the evening drinking tea, chatting and observing late-night activities, i.e., where people go to sleep, women preparing food for the next day, and people visiting each other to discuss important matters after work. Daytime participant observations included accompanying adults and children during their routine activities such as fetching water, and going to the farm and cattle herds. During these activities informal conversations were conducted in English and Fula by the main author and in English, Fula, Wolof, and Mandinka by the research assistants. These informal conversations were written down after the interview as soon as appropriate. The fieldworkers were trained on how to note down observations and informal conversations and were very closely supervised during the research process. In total 12 observations and 22 informal conversations were formally written up.

**In-depth interviews** were conducted at participants’ residences in confidentiality or in places where they felt most at ease. When necessary, to overcome language barriers, the interviews were carried out with the assistance of trained field assistants. Most interviews were recorded; however, if the interviewee preferred not to be recorded, the responses were written down in detail during the interview. A total of 176 interviews were conducted, transcribed and translated into English. 79 interviewees were female of which 64 stated that their occupation was farming or gardening and housework. Other occupations included herding (2), petty trade (2), traditional birth attendant (3), teaching (1), and weaving (1). Four women were elderly and stated that they were no longer working; Ten research participants were adolescents whose main occupation was housework. The total number of male interviewees was 97, of which 69 stated that their occupation was farming. Other occupations included herding (13), business (4), marabouts (6), herbalist (1), Imam (1), Arabic teacher (1), school-chairman (1), policeman (1), painter (1), builder (1), labourer (1), griot (1), apprentice for transport (1), secondary school pupil (1).

**Group discussions** were conducted when informants agreed to be interviewed together or to get different views on a particular topic. In total, four group discussions were conducted: two of these discussions were held with male and female MRC fieldworkers on witchcraft, supernatural beliefs and the meaning of blood; two FGDs were held with women belonging to the status groups of former slaves.

### Sampling

Sampling was purposive following the principles of ethnographic data collection [27]. The research participants were (i) identified during participant observation and informal conversations or (ii) selected on the basis of relevant analytical categories (such as gender, age, religion, ethnicity, social standing, role within the community) which were identified during fieldwork. Maximum variation in the selection of respondents ensured that all social groups were represented, regardless of local hierarchy or locally perceived expertise. Critical cases (informants that presented with significant or unusual findings) were continuously included in the sampling frame until saturation was reached. Snowball sampling was used to enhance participants’ trust and confidence in the research team when information was needed.

### Data analysis

At first, relevant categories for analysis (social realities) were established during the field research. This was an iterative, concurrent and exploratory process. Once analytic categories relevant to the research question emerged, the results were confirmed and constantly tested for their validity by including critical cases. The verification and validation of the results took place by systematically interviewing people belonging to different analytic categories on the same topic (e.g., men *vs* women, high *vs* low status groups, etc.). Once different analytic categories were confirmed and data saturation was reached, the results were further analysed and categorized after the transcription process.

### Ethical considerations

In the Gambia, the study was approved by the MRC Scientific Coordinating Committee and by the Gambia Government/MRC joint Ethics Committee (SCC number 1351). The study was also approved by the Institutional Review Board (IRB) of the Institute of Tropical Medicine, Antwerp, Belgium. The interviewers followed the Code of Ethics of the American Anthropological Association (AAA). All interviewees were informed of the study, the type of questions to be asked and the intended use of the results prior to the interview. Anonymity and confidentiality were guaranteed and they were informed that they had the right to refuse or stop the interview at any time. Verbal instead of written consent was preferred as requesting the subject’s signature could have been a potential reason for mistrust. A witness acknowledged the verbal consent and signed a documentation of consent sheet. Both ethics committees approved the oral consent procedure.

## Results

### Local definitions of uncomplicated malaria

The term ‘malaria’ was considered to be a ‘*toubab’* (meaning ‘white man’) word – referring to the biomedical doctors’ expression – for a sickness locally referred to as *Kadjeh*, *Sainaabo, Fula Kajeo* or *Susula Kurango* in Mandinka, *Jontinooje* or *Kajeje* in Fula, *Samama Ntong Yea* in Soninke (Serahule), and *Sibirru* in Wolof. Respondents unanimously described malaria as the disease that occurs during the rainy season, the period “when the maize ripens” and “the period when people are busy working on the farm” (October-December).

The term ‘malaria’ was described as an illness with a combination of some of the following symptoms: fever, the person feels cold even though their body is hot, severe headache, continuous vomiting of yellowish or greenish colour (“the colour of maize” or “leaves”), lack of appetite, fatigue, and inability to work. At times additional signs were mentioned such as “yellow eyes” and “the urine turning red”. Middle-aged or elderly individuals would use ‘malaria’ for any condition of tiredness, fever and vomiting in combination with any other of the above-mentioned symptoms, with no mention of diagnostic tests. Conversely, among younger people, many stated that only a rapid diagnostic test (RDT) test could confirm whether the sickness was actually malaria.

Although many people equated malaria to their local ‘translations’, sometimes these were perceived to be different diseases to malaria, but with similar symptoms (Table 1). For example, in certain North Bank study sites (Upper River Region) study participants frequently reported that malaria was a very serious disease caused by mosquitoes but could be easily treated at the health centre, whereas *Jontinooje/Kajeje* (malaria in Fula) was a very common disease of unknown cause.

**Table 1:** **Quotes illustrating perceptions of different disease categories**

**Perceptions**	**Respondent**
*I: Is Malaria the same as Jontinooje and Kajeje?*	Young woman, farmer; Fula, Sahre Sillere
R: They are different.	
*I: What makes them different?*	
R: Malaria is caused by mosquitoes whilst *Jontinooje* and *Kajeje* comes at the time when maize starts growing.	
*I: How does it make people sick, is it when you eat the maize or?*	
R: We only know that when maize starts growing, that is the time people get sick with *Jontinooje*.	
*I: So you don’t know how it gets into the body?*	
R: No I don’t.	
*I: Is Malaria the same as Kajeje and Jontinooje or are they different diseases?*	Young woman, farmer; Fula, Sahre Sillere
R: They are different.	
*I: What makes them different?*	
R: *Kajeje* is a disease that comes at a particular time. When it’s time comes, people get sick with it. For malaria, it is caused by mosquitoes. When mosquitoes bite you, you will get sick with malaria and your body will become very hot and you will feel cold. You will be sick for a while, then recover.	
*I: What is the difference (between Malaria, Jontinooje and Kajeje)?*	Young man, teacher; Fula Sahre Sillere
R: For *Fula Kajeje* and *Jontinooje*, they affect people mainly in the dry season whilst people get sick with malaria during the raining season when we have lots of mosquitoes around. You don’t see mosquitoes in our area here during the dry season but immediately when the rainy season commences, people will start getting sick with malaria. (…) With *Jontinooje* today you will feel sick and you will recover the next day, get sick again the next and recover the following day. So that is how *Jontinooje* rotates. For *Kajeje* you will get sick at night and recover in the morning. (…)	
*I: What causes Jontinooje and Kajeje?*	
R: No I don’t really know what causes them but I know it affects people after the rainy season.	

### Perceived aetiology of malaria

#### General cosmology

According to local Gambian cosmology, sickness is considered to be caused by either: i) an agent or micro-organism inside the body that is detectable by biomedicine (such diseases are called *kuraŋ keso* in Mandinka); or, ii) by supernatural forces, originating “outside the body”, that are undetectable through biomedical diagnosis (called *ming kesa sande*). Examples of these are “foul wind” (*fonyo jawo*) and illnesses caused by *Jinne* spirits or witchcraft. This classification directly affects consequent treatment-seeking itineraries. In addition, several perceived causes may co-exist or inter-relate and require a combination of treatments.

### Uncomplicated malaria

Uncomplicated malaria is categorized as *kuraŋ keso*. When participants were asked what the cause of malaria was, almost all responded that mosquitoes were involved in the sickness. However, mosquitoes were not believed to be the only cause of malaria. Further singular causalities were: i) climate-related factors such as the cold (“the cold coming from the ground when you don’t wear shoes”; “when the cold wind blows and you are sleeping outside”); ii) infection, as in direct contact with other infected people (i.e., sharing a calabash with someone who has malaria, sleeping next to someone who has malaria, transmission from animals); iii) hygiene (dirty hands before eating food); iv) certain foods such as sour milk, sorrel (hibiscus) that is not cooked properly, oily food, “jumbo” food flavouring; and, v) malnutrition. Those who did not think that malaria was the same as local illness categories (e.g. *Jontinooje*, *Kajeje*) said that they did not know what the cause of the disease was (Table 1).

### Severe malaria

For symptoms suggesting severe malaria, such as convulsions, fainting, disorientation, and confusion, the illness was often attributed to the affliction of supernatural entities or factors “outside the body” (*ming kesa sande),* such as *Jinne* or witches.

***Jinne*** are non-human spirit creatures that are believed to inhabit the world alongside humans. All questioned participants believed in them and, because they are mentioned in the Holy Koran, their existence is indisputable for local Muslims. *Jinne* are said to be invisible to humans under ‘normal’ circumstances and do no harm although some ‘bad’ *Jinne* can afflict human beings, causing aggressive behaviour, ‘madness’ and mental illness, sickness or death by ‘attacking’ a person through ‘wind’ or in ‘unsafe’ places in the bush. *Jinne* are commonly believed to dwell in trees in the bush and attack specifically vulnerable people, e.g., pregnant women walking through their territory. However, a *Jinne* may attack anywhere and no place is completely safe. Most of the symptoms described match the clinical presentation of severe malaria or other serious diseases, such as meningitis or septicaemia (Table 2). Mentally unstable or developmentally disabled individuals (including children) are believed to be possessed by *Jinne*. Their state of mind is said to depend on the *Jinne’s* moods and the extent to which the marabout’s treatment is driving the force of the *Jinne* out of the body. Marabouts are well-respected religious figures across West Africa who specialise in Islamic and non-islamic spiritual matters such as prayers, healing, clairvoyance and other rituals around life and death.

**Table 2:** **Quotes illustrating perceived symptoms of** ***Jinne*** **attacks**

**Perceived symptoms**	**Respondent**
For the *Jinne* if it attacks a person will be shouting and collapsing up and down, and will sometimes sleep and at times wake up and be aggressive.	Middle-aged man, Imam; Wolof, Boiram
They can make an individual to fall unconscious, if it wants to disturb you most it makes you collapse totally. Some will go to the marabouts and take things to take bath, all those are ways of protecting oneself against the *Jinne*.	Middle-aged woman, farmer; Fula, Keneba
They (*Jinne*) can also cause it (malaria) in a way like if you have contact with the air that blows from them then that can also cause it. Sleeping outside can also give an individual some troubles because if you do so, you can come into contact with some other things. (…) When their air touches you while you were not aware of it, that can also cause you harm.	Young woman, farmer; Mandinka, Niji
*I: Now, if an individual is attacked by the* Jinne *what are the symptoms that he shows?*	Middle-aged woman, farmer; Mandinka Niji
R: It has different signs; some will get unconscious, and will start doing some other dubious things. Then people will start saying that let us try the traditional means to see whether the person has been attacked by the ‘bad wind’.	
What the *Jinne* can do is that they will twist your mind upside down; it makes some collapse and fall unconscious, they can also do that. They make some people mad who will then just be wandering on the streets and making false utterances that are beyond the comprehension of a conscious person.	Old man, retired; Mandinka Niji
They (*Jinne*) are found in the bush; you know there is a time that is not safe for an individual to go to the bush. If you go to the bush during such times, you are liable to be attacked by the *Jinne*.	Middle-aged woman, farmer; Mandinka, Niji

**Witchcraft** is another malaria-related aetiology. Whereas *Jinne* are non-human creatures, *buwaa* or *sukuñaabe* are humans that metaphorically ‘eat’ other human beings. In English, Gambians mostly call them ‘witches’. The *buwaa* possess a quality that makes them addicted to spiritually consuming other human beings – ‘sucking out’ their life force until the victims get very sick and die. A Wolof marabout explained this process the following way: “You know if you put the insect in a small container with a groundnut, it will suck away all the liquid contained in the seed. That is the same way that the witches operate”. Witches are said to “like to get involved” when someone has malaria and “finish off” the sick person. The symptoms someone experiences when attacked by a witch tend to be mainly described as rib pain (Table 3). Most respondents, however, say they do not know how witches operate and how they eat people. An experienced marabout explained that “witches have two different kinds of eyes, the eyes of darkness and the eyes of day light – just like a normal human being. When you place an opaque bottle here and put something inside, the witch will see through and know what it contains. Before it attacks a person, it will see all the organs that are in the human body […] that is the reason why if you are with a person who is suspected of being a witch, don’t allow the person to be at your back, instead ask the person to stay in front and then follow him so that it will not see through you.”

**Table 3:** **Quotes illustrating perceived symptoms of witchcraft (** ***Buwaa*** **) attack**

**Perceived symptoms**	**Respondent**
When you are being attacked by witchcraft, it gives you rib pain and eats all the organs that are in the stomach’	Young woman, housework; Fula, Boiram
For some people, if a *Buwaa* follows a person, it gives him a pain in the ribs. If that happens then they will start saying that Mr X has rib pain and let us do something about it because rib pain is mostly associated with the issue of these black forces.	Young woman, housework; Mandinka, Niji
For the *Buwaa*, if they attack you, you keep on lying down and if God does not help, you will die. At times, if the person takes some herbs then he/she gets better.	Old man, retired; Mandinka, Niji
Those sicknesses are there. If someone is being attacked by *Buwaa,* in most cases, the ribs will start paining. Those are the signs. We called them the ‘night people’.	Middle-aged man, herder; Fula Sahre Gella
*I: What are the symptoms of a person affected by a Buwaa?*	Middle-aged woman, farmer; Fula, Sahre Gella
R: For us here, so many children get affected and one of my children is among them. Once two *toubabs* (white people) came to this village and I took them to my house to show them my sick child but they asked me to take him to the hospital which I did. He got some medication but the treatment didn’t work so I decided to go to a marabout. And thanks to God he gained his health back.	
*I: What symptoms did he show?*	
R: He was always trembling and sometimes collapsed until we poured some water on him.	

The above-mentioned supernatural entities are said to cause *ming kesa sande* sicknesses, and are believed to commonly afflict individuals through “foul wind” (*fonyo jawo* in Mandinka and *heendu bonndu* in Fula). Gambians speak of foul wind in an unspecific way when it is unknown what kind of creature (*Jinne* or witch or other) is believed to have caused sickness (e.g., malaria).

### The diagnosis and treatment of uncomplicated malaria

Different diagnostic methods of detecting malaria and, consequently, perceptions of who is capable of effectively doing so, co-exist. Participants who thought that an individual had malaria as soon as they had symptoms of tiredness, severe headaches and vomiting, believed that the best treatment was biomedical and it would be necessary to attend a health facility. Some individuals, particularly the older ones (65–90 years of age), would consume natural treatments prepared from tree bark as soon as they felt “malaria was coming on”. However, they still felt that biomedical treatment was more effective as it got rid of the symptoms “within three days”. Those advocating RDTs as the only reliable diagnostic method also believed in biomedical treatment.

### Biomedical diagnosis and treatment

Health facilities were perceived to effectively diagnose and treat illnesses that were understood to be caused by small infectious organisms in the body (*kuraŋ keso* illnesses). If a person was thought to have ‘malaria’ a visit at the health facility was thought to be required for diagnosis and treatment.

### Traditional treatment

‘Herbal treatment’ or ‘trees’ (*leɗɗe ɓaleeɓe, garab*) were often consumed as first emergency treatment for sicknesses involving vomiting, nausea, diarrhoea, stomach pain, until funds had been raised to take the sick person to the health centre. The middle-aged (35–65 years of age) and older generations (65–90 years of age) were used to consuming natural treatments for weakness and tiredness. It was common to put the leaves of some trees or bushes in the bathwater to refresh the body when suffering from tiredness. In all households there were individuals who could point out where in the neighbourhood their source of healing trees or bushes were positioned. The advantage of using herbal treatment or ‘trees’ was that it was free and readily available at all times without having to consult a doctor or marabout. However, younger respondents (15–35 years of age) stated they did not like the taste of these trees and preferred to attend a health facility or buy paracetamol. Almost all respondents said that they stopped consuming herbal treatments as soon as they had been prescribed biomedical treatments.

In cases when malaria was not perceived to be the same as the local folk illnesses (i.e. *Kajeje* or *Jontinooje*, often translated as ‘malaria’), respondents would not rush to the health centre for diagnosis or treatment. They would only go if they thought that they were suffering from something more serious (e.g., malaria) or if the symptoms were getting more severe. They reported almost exclusively drinking tea made of the leaves of trees (particularly *neem* tree and *neverdie*) when suffering from *Jontinooje/Kajeje* (Table 1).

### Access and affordability of biomedical treatment

An important reason for delay in treatment is related to the lack of financial resources to travel to a health facility. Household heads in charge of financial resources were reported to not make money for treatment available when a person was perceived to suffer from a ‘mild’ ailment that may go away without treatment. It was not until a person was perceived to be moderately to severely sick, i.e., if the patient was bed-ridden and no longer able to work, that resources for transport to the health centre or hospital were made available or borrowed. Respondents reported to rely on herbal treatment as a first remedy for malaria while looking for the means to take the sick person to the health centre.

### The diagnosis of severe malaria

If a sick person was suffering from severe malaria involving hallucinations and unpredictable behaviour, respondents agreed that these symptoms could be caused by supernatural entities (witchcraft or *Jinne*) or foul wind and, therefore, it was important to consult a marabout for diagnosis (Tables 2, 3 and 4). The marabouts’ methods of diagnosis are based on divination practices using either the Islamic practice “*listahar”* or “black magic”. Both Islamic and “black magic” diagnoses are referred to non-specifically as *timgal* in Fula and *jeebero* in Mandinka, which is interpreted as “to check yourself out” or ‘diagnosis’*.* In general people believe that most sicknesses or misfortunes are due to spiritual blockages. Individuals suffering from fever and hallucinations are thus believed to be suffering from a spiritual blockage inflicted by a *Jinne* or a witch or the evil deeds (black magic) of another person. If the individual is seriously sick, it is crucial to identify what exactly has caused the blockage before the person can recover. The correct treatment depends on the correct diagnosis by a marabout. This means that a person suffering from severe malaria may not consult a biomedical health practitioner because he/she believes that a spiritual blockage inflicted by a *Jinne* or witchcraft is causing the sickness – a case for a marabout.

**Table 4:** **Quotes illustrating perceived optimal treatments for malaria**

**Perceived optimal treatments**	**Respondent**
Malaria should not be treated through the traditional line. The medical treatment is the most effective method because they will examine my body and give me medications to take.	Young woman, housework; Mandinka, Niji
The only way that I recommend is the clinical treatment. If my child gets infected I rush him to the clinic and with the help of God, it will get okay within a short period of time even before the medicine gets finished.	Elderly woman, farmer; Mandinka, Niji
I prefer the clinical method of treatment because they are the ones who can be able to see and diagnose a disease that I cannot myself. When they examine my body they know exactly what part of my body is infected. Because the traditional way of treatment cannot help you enough to know what you are actually suffering from.	Young Man, business; Mandinka, Kulluh Kouleh
The pharmacy is very expensive so that we normally do not go because we do not have money. So if we do not have the money we use our traditional medicines like leaves and herbs.	Middle aged woman, farmer; Fula, Sahre Sillere
If those are the causes (*ming kesa sande*) then we should try the traditional treatment to see because the medical line would not be able to cure such diseases. If at all the sickness is caused by mosquitoes only, then the hospital will be able to do something about it but if these forces get themselves involved, the hospital cannot cure that.	Young woman, farmer; Mandinka, Niji
When one is sick with *Kajeje*, we use *haako duke* (leaf) and hit the sick person on the head for seven times then allow the leaf to dry in the sun, as soon as the leave gets dry, they sick person will recover. Or we also put coos (millet) on the person’s head for some time so that when the person vomits a greenish colour, he/she will stop vomiting after the coos has been put on the person’s head.	Elderly man, retired herder; Fula, Sahre Sillere
Sometimes people come here with herbs, put them in water, boil it and ask you to drink it. Or they will also take you to the hospital, test your blood, so when you have malaria they will give you the required treatment.	Young man, teacher; Fula, Sahre Sillere

The Islamic *listahar* is the most conventional method to diagnose blockages that does not involve black forces. The marabout recites some prayers at night that inspire a dream in which God lets the marabout know the cause of the disease and what remedies can cure the person. Only a marabout with good knowledge of the Koran can use this method. Although this is the most conventional method there are many other traditional non-Islamic forms of divination to diagnose (*timgal*/*jeebero*) ‘blockages’, such as marking lines on a piece of paper or in the sand to diagnose witchcraft. It is also common to throw cowrie shells, do palm-readings, use a calabash filled with clean water and throw eight short sticks into the water while reciting incantations and talking to the consulting person about their problems based on the movement of the sticks in the water. None of the marabout respondents ever physically examined their patients.

### Spiritual treatment for severe malaria

The treatment given depends on the marabout’s family traditions and common practices. Some marabouts use herbs from the bush while others just tell their patient what charities they have to give. Once the marabout knows which Koranic verses will help the person through the performance of *listahar*, he writes those verses on a blackboard or a piece of paper. If the verses are written on a blackboard, the marabout wipes them off with a cloth and instructs the sick person to put the cloth in a bottle of water with perfume. If the verses are written on a piece of paper it is dissolved in the bottle with perfume. The person is instructed to wash their body with such water twice a day for a recommended period of time. The washing is believed to apply the needed Koranic verses onto the body and remove the ‘blockages’ that are making the person sick (e.g., the *Jinne* possessing the body or the soul-eater spiritually devouring the flesh). It is also common to sew the piece of paper with the Koranic verses into a leather amulet to be worn at all times.

## Discussion

Beliefs of the cause of illness influence treatment-seeking decisions. At first glance, these results show that the majority of people seek biomedical treatment for uncomplicated malaria. These findings are in line with the research of Clarke *et al.* in the Gambia, who found that 63% of respondents took their children to the health centre for malaria treatment and 13% to a health village post [24]. Wiseman *et al*. found that 48.6% of respondents went to the hospital for malaria treatment, 21.7% to the health centre and 4.5% to the clinic [29]. These findings would suggest that five or ten years ago at least 24% did not go to the health centre when they thought they had ‘malaria’.

However, further evidence calls into question the apparently high number of people who seek treatment at health centres. Certain folk illnesses, such as *Jontinooje* and *Kajeje,* are not considered to be malaria by part of the population but as self-limiting febrile illnesses – consequently not leading to treatment-seeking in the biomedical sector. These illnesses are often translated as malaria, although they epistemologically do not correspond to the theoretical biomedical model of malaria. The existence of these folk illnesses and consequent implications are generally ignored in malaria research and in public health practice. When health providers talk to patients about *Jontinooje* to facilitate communication by adapting to the local language, they are unwillingly basing their explanations, health-care messages and instructions on a different conceptual framework than the one the patient is using. Thus, the systematic equation of certain folk illnesses with ‘malaria’, without further explanation of disease aetiology, may create delay in treatment-seeking. Dugas *et al.* reported a similar situation in Burkina Faso and warned of the conceptual mismatch when translating ‘malaria’ (biomedical epistemology) to *sumuya*, which has a different aetiology in the traditional medicine epistemology [30].

In addition to misunderstandings due to overlapping conceptions of folk illnesses, the interpretation of symptoms can also become problematic. When malaria cases present with symptoms such as convulsions, hallucinations and/or loss of consciousness, found for example in patients with severe malaria, the illness is often interpreted as having a supernatural aetiology. As a consequence, a traditional healer is consulted for diagnosis and treatment. Literature from a wide variety of sub-Saharan African countries presents a similar association between symptoms of severe malaria and spirit attacks [15,17-22]. Although beliefs in spirits, witches and *maraboutage* are well documented in the Senegambia region [30-36], there is a lack of ethnographic and sociobehavioural research describing local beliefs of sickness caused by supernatural entities, their relation to malaria or other febrile illnesses and consequent health-seeking behaviour in the Gambia. Madge describes the use of herbal medicine among the Jola in the Gambia but, contrary to the findings of the research presented in the results section, she suggests that marabouts’ treatments for diseases afflicted by spirits are only preventive, not curative [37]. This is relevant, especially when considering that the use of these traditional treatments for severe malaria may delay life-saving treatment.

An important reason for the use of alternative treatments is to overcome the time while resources are sought to cope with the disease. Because of the cost burden, a patient is often not taken to the health centre or hospital until moderately to severely sick. Data on the socio-economic impact of malaria on households in the Gambia is rather scanty. A study in 2006 on patterns of expenditure for malaria prevention in the rural town of Farafenni in the North Bank Region, reported an average household expenditure of US$1.6 per month for buying malaria protection products, about 10% of the average monthly earnings of a subsistence farmer [38]. Since then inflation has affected the Gambian economy further, aggravating the financial burden on rural farmers whose main income consists of subsistence farming and herding. More recent figures of monthly expenditures on malaria protection products, treatment and transport to health facilities are not available.

Delay in treatment, or the absence of it, due to symptom misinterpretation, perceived low severity of risk, or cost burden is problematic from a public health point of view. Recent research is concerned with identifying how, when and through whom malaria transmission is maintained and molecular tests have shown that the asymptomatic parasitic reservoir is common, even in low-endemic areas [39,40] such as the Gambia. This qualitative study suggests that even patients with clinical malaria, who do not recognize the symptoms as such, or who do not perceive them as a serious threat to their health, may represent an additional reservoir of infection as they are not reached.

The strength of this study lies in its in-depth understanding of how malaria symptoms can be interpreted as different disease categories and thus attributed to different causes, leading to different health-seeking itineraries, even when an individual knows that ‘malaria’ is transmitted through mosquitoes and what the biomedically prescribed treatment regime is. A limitation of this study is the lack of quantitative malariometric data to directly relate different disease categories to malaria infection. This was, however, outside of the scope of the study.

## Conclusion

The data presented shows that healthcare professionals and researchers need to take local disease categories and folk illnesses seriously and adapt their responses accordingly, as the inability to respond effectively leads patients to seek treatment outside the health sector. Furthermore, it is crucial that medical professionals doing consultations in local languages continue to explain the cause of malaria and address beliefs in local illness categories such as *Joninooje*, *Kajeje*, *Susula Kurango* or any other name given to malaria symptoms that require biomedical treatment.
